# Medulloblastomas with *ELP1* pathogenic variants: A weakly penetrant syndrome with a restricted spectrum in a limited age window

**DOI:** 10.1093/noajnl/vdae075

**Published:** 2024-05-15

**Authors:** Léa Guerrini-Rousseau, Julien Masliah-Planchon, Mathilde Filser, Arnault Tauziède-Espariat, Natacha Entz-Werle, Christine M Maugard, Saskia M J Hopman, Jacob Torrejon, Marion Gauthier-Villars, Fatoumata Simaga, Thomas Blauwblomme, Kevin Beccaria, Etienne Rouleau, Marina Dimaria, Jacques Grill, Samuel Abbou, Béatrice Claret, Laurence Brugières, François Doz, Yassine Bouchoucha, Cécile Faure-Conter, Valerie Bonadona, Ludovic Mansuy, Emilie de Carli, Olivier Ingster, Clémentine Legrand, Anne Pagnier, Pascaline Berthet, Damien Bodet, Sophie Julia, Anne-Isabelle Bertozzi, Marjolaine Wilems, Claude-Alain Maurage, Olivier Delattre, Olivier Ayrault, Christelle Dufour, Franck Bourdeaut

**Affiliations:** Molecular Predictors and New Targets in Oncology, Inserm U981 Team “Genomics and Oncogenesis of Pediatric Brain Tumors,” Gustave Roussy, Université Paris-Saclay, Villejuif, France; Department of Children and Adolescents Oncology, Gustave Roussy, Université Paris-Saclay, Villejuif, France; Department of Pathology and Diagnostic, Prognostic and Theranostic Medicine, Somatic Genetic Unit, Institut Curie, Paris Sciences Lettres Research University, Paris, France; Department of Pathology and Diagnostic, Prognostic and Theranostic Medicine, Somatic Genetic Unit, Institut Curie, Paris Sciences Lettres Research University, Paris, France; Department of Neuropathology, Sainte Anne Hospital, Paris, France; Pediatric Hematology and Oncology Department, Strasbourg University Hospital, University of Strasbourg, Strasbourg, France; Department of Clinical Genetics, Strasbourg University Hospital, Strasbourg, France; Department of Genetics, University Medical Center Utrecht, Utrecht, the Netherlands; Université Paris Sud, Université Paris-Saclay, CNRS UMR 3347, INSERM U1021, Orsay, France; Institut Curie, Paris Sciences Lettres Research University, CNRS UMR, INSERM, Orsay, France; Department of Genetics, Institut Curie, Paris Sciences Lettres Research University, Paris, France; Department of Genetics, Institut Curie, Paris Sciences Lettres Research University, Paris, France; Necker-Enfants Malades University Hospital, Department of Pediatric Neurosurgery, Paris-Cité University, Paris, France; Necker-Enfants Malades University Hospital, Department of Pediatric Neurosurgery, Paris-Cité University, Paris, France; Cancer Genetics Unit, Department of Biology and Pathology, Institut Gustave Roussy, Villejuif, France; Department of Children and Adolescents Oncology, Gustave Roussy, Université Paris-Saclay, Villejuif, France; Molecular Predictors and New Targets in Oncology, Inserm U981 Team “Genomics and Oncogenesis of Pediatric Brain Tumors,” Gustave Roussy, Université Paris-Saclay, Villejuif, France; Department of Children and Adolescents Oncology, Gustave Roussy, Université Paris-Saclay, Villejuif, France; Department of Children and Adolescents Oncology, Gustave Roussy, Université Paris-Saclay, Villejuif, France; National Institute for Health and Medical Research (INSERM) U1015, Gustave Roussy, Villejuif, France; Psycho-Oncology Unit, Interdisciplinary Department of Supportive Care, Gustave Roussy, Université Paris-Saclay, Villejuif, France; Department of Children and Adolescents Oncology, Gustave Roussy, Université Paris-Saclay, Villejuif, France; Université Paris Cité, SIREDO Pediatric Cancer Center, Institut Curie, Paris, France; SIREDO Center (Care, Innovation Research in Pediatric, Adolescent and Young Adult Oncology), Institut Curie, Paris, France; SIREDO Center (Care, Innovation Research in Pediatric, Adolescent and Young Adult Oncology), Institut Curie, Paris, France; Pediatric Hematology and Oncology Institut, Centre Léon Berard, Lyon, France; Clinical Oncogenetics Unit, Department of Prevention and Public Health, Centre Léon Bérard, Lyon, France; Department of Pediatric Hematology and Oncology, Centre Hospitalo-Universitaire de Nancy, Vandœuvre-lès-Nancy, France; Pediatric Hematology and Oncology Department, Angers University Hospital, Nancy, France; Department of Genetics, Angers University Hospital, Angers, France; Department of Genetics, Grenoble University Hospital, Grenoble, France; Department of Pediatric Hematology and Oncology, Centre Hospitalo-Universitaire de Grenoble, Grenoble, France; Department of Genetics, Centre François Baclesse, Caen, France; Pediatric Hematology and Oncology Department, Caen University Hospital, Caen, France; Department of Genetics, Toulouse University Hospital, Toulouse, France; Pediatric Hematology and Oncology Department, Toulouse University Hospital, Toulouse, France; Department of Medical Genetics, Montpellier University Hospital, Institute for Neurosciences of Montpellier, Univ Montpellier, INSERM, Montpellier, France; Laboratory of Neuropathology, Centre Oscar Lambret, Lille, France; INSERM U830, Cancer, Heterogeneity, Instability and Plasticity Laboratory Institut Curie, Paris, France; Université Paris Sud, Université Paris-Saclay, CNRS UMR 3347, INSERM U1021, Orsay, France; Institut Curie, Paris Sciences Lettres Research University, CNRS UMR, INSERM, Orsay, France; Molecular Predictors and New Targets in Oncology, Inserm U981 Team “Genomics and Oncogenesis of Pediatric Brain Tumors,” Gustave Roussy, Université Paris-Saclay, Villejuif, France; Department of Children and Adolescents Oncology, Gustave Roussy, Université Paris-Saclay, Villejuif, France; Université Paris Cité, SIREDO Pediatric Cancer Center, Institut Curie, Paris, France; SIREDO Center (Care, Innovation Research in Pediatric, Adolescent and Young Adult Oncology), Institut Curie, Paris, France; INSERM U830, Cancer, Heterogeneity, Instability and Plasticity Laboratory Institut Curie, Paris, France

**Keywords:** cancer predisposition syndrome, ELP1, medulloblastoma, pathogenic variant

## Abstract

**Background:**

*ELP1* pathogenic variants (PV) have been recently identified as the most frequent variants predisposing to Sonic Hedgehog (SHH) medulloblastomas (MB); however, guidelines are still lacking for genetic counseling in this new syndrome.

**Methods:**

We retrospectively reviewed clinical and genetic data of a French series of 29 *ELP1*-mutated MB.

**Results:**

All patients developed SHH-MB, with a biallelic inactivation of *PTCH1* found in 24 tumors. Other recurrent alterations encompassed the *TP53* pathway and activation of *MYCN/MYCL* signaling. The median age at diagnosis was 7.3 years (range: 3–14). *ELP1*-mutated MB behave as sporadic cases, with similar distribution within clinical and molecular risk groups and similar outcomes (5 y – OS = 86%); no unusual side effect of treatments was noticed. Remarkably, a germline *ELP1* PV was identified in all patients with available constitutional DNA (*n* = 26); moreover, all tested familial trio (*n* = 11) revealed that the PVs were inherited. Two of the 26 index cases from the French series had a family history of MB; pedigrees from these patients and from 1 additional Dutch family suggested a weak penetrance. Apart from MB, no cancer was associated with *ELP1* PVs; second tumors reported in 4 patients occurred within the irradiation fields, in the usual time-lapse for expected radiotherapy-induced neoplasms.

**Conclusions:**

The low penetrance, the “at risk’ age window limited to childhood and the narrow tumor spectrum, question the actual benefit of genetic screening in these patients and their family. Our results suggest restricting *ELP1* germline sequencing to patients with SHH-MB, depending on the parents” request.

**Key Points**

- *ELP1*-mutated MBs behave as sporadic cases.
- Allsomatic *ELP1* PVs were inherited, with a low penetrance for MB onset and no other tumor risk.
- We questioned the benefit of genetic screening in *EPL1*-mutated MB patients and their families.

**Importance of the Study**

*ELP1*-related predisposition syndrome has been recently described and is now looked for in clinical practice in patients with Sonic Hedgehog subtype medulloblastomas. However, the actual impact of finding a pathogenic variant (PV) in *ELP1* in terms of genetic counseling is largely undetermined, since the available data don’t bring sufficient details to fully address the penetrance or the tumor spectrum issues. Here, we report on a large national series and describe the behavior of the tumors, the tolerance of the treatment, the incidence of germline PV, and the inheritance pattern. We thereby bring useful information to help decide whether screening for *ELP1* PV will be of clinical utility for patients and their families.

Large-scale genome-wide sequencings performed within the last decade suggest that up to 10% of pediatric malignancies are related to a genetic predisposition syndrome.^1^ Among those malignancies, medulloblastomas (MB) are rare embryonal tumors developing from various cerebellar embryonal progenitors, consensually categorized in 4 different molecular entities defined according to their cell of origin and their main oncogenic drivers, ie WNT, SHH *TP53*-wildtype, SHH *TP53*-mutant, and non-WNT/non-SHH^2^ (including MB-group 3 and MB-group 4). In a recent study investigating the germline DNA of over 1000 patients affected with MB during childhood, Waszak et al. identified 6 cancer predisposition genes (CPG) likely related to MB occurrence, accounting for about 5% of all patients with MB.^3^ Of note, cancer predisposition syndromes (CPS) are mainly found in the MB-SHH group, with up to 20% of patients baring a predisposing pathogenic variant (PV) in this group.^3^ Historically, MB-SHH was first related to Gorlin syndrome, a genetic condition caused by germline heterozygous PV in *PTCH1*^4^; further studies eventually enlarged the definition of Gorlin syndrome to patients baring a germline PV in *SUFU*, which acts together with *PTCH1* as a repressor of SHH signaling, and is also increasing the risk of MB-SHH.^5^ The cumulative lifetime risk of developing MB is now considered to be <1% and about 15% in *PTCH1* and *SUFU* PV carriers, respectively.^6,7^ More recently, PV in *GPR161* were discovered as another, extremely rare, MB predisposing syndrome.^8^ Similarly with *SUFU* and *PTCH1*, *GRP161* PV predispose to early MB onset, before 5 years of age.^7,9^ On the contrary, later-onset MB-SHH was rather related to *TP53* PV, and MB-SHH in older children, teenagers and young adults may be the entry point to the discovery of Li–Fraumeni syndrome.^10^*TP53*-mutated MB-SHH are now known to be a highly devastating disease.^11^ Finally, Waszak et al. identified PV in *ELP1* in up to 15% of pediatric MB-SHH patients, which makes *ELP1*-related CPS the most frequent in MB patients.^12^

*ELP1* gene, which is located in the 9q31.3, 13Mb from *PTCH1* locus (9q22.3), encodes for ELP1 protein, a component of the elongator complex which comprises 6 subunits (ELP1–6) and is implicated in neurogenesis.^13,14^ Remarkably, *ELP1* PV systematically co-occur with *PTCH1* biallelic inactivation in tumors, following the so-called “four hits-three steps” model, which strongly suggests that ELP1-deficiency predisposes to tumor development in combination with constitutive activation of SHH signaling. On the opposite, *ELP1* PVs have been described to be mutually exclusive with germline and somatic *TP53* mutations.^12^

So far, given the limited number of publications reporting the phenotype and outcomes of *ELP1* germline PV carriers, the actual penetrance and cancer spectrum of this emerging CPS remain uncertain, which precludes adapted recommendations for both testing asymptomatic relatives and tumor surveillance in PV carriers. Here, we present our experience at the French national level with patients affected by MB with *ELP1* PV in order to increase knowledge and better adapt genetic counseling in this not-so-rare genetic condition.

## Patients and Methods

### Identification of ELP1 PV in MB Samples

MB samples were identified firstly from the tumor collection of the Unite de Génétique Somatique (UGS) at Institut Curie, which collects frozen MB samples at the national level for molecular profiling within the clinical routine and from 2005, and secondly from the tumor collection of Sainte-Anne and Necker-Enfants Malades (NEM) hospitals, in Paris, which collects frozen and formalin-fixed paraffin embedded (FFPE) samples for all MB resected at the pediatric neurosurgery department of NEM Hospital. For FFPE MB samples from the latter collection, immunostaining using an anti-ELP1 antibody was used as described in Tauziede-Espariat et al.^15^ and allowed the identification of 12 ELP1-deficient MB, which were subsequently sequenced. For tumors identified in the UGS collection, DNA was extracted according to classical procedures and sequenced according to Tauziede-Espariat et al.^15^ The library was prepared with the SureSelect XT-HS according to the manufacturer’s protocol (Agilent) and sequenced on an Illumina NovaSeq 6000. The sequences of all coding exons of *ELP1* (NM_003640.4) were analyzed afterwards.

The WES on matched tumor/constitutional DNA for 5 patients (MB07_04, MB08_02, MB08_15, MB15_04, and MB15_12), was carried out using the German Cancer Research Center and European Molecular Biology Laboratory (DKFZ and EMBL) cancer genome analysis pipelines in accordance with ICGC PCAWG (https://dcc.icgc.org/pcawg). More details are reported in the princeps article.^12^ The samples are included in the MB Comics cohort and the study was approved by the Institutional Clinical Research Board of Gustave Roussy and complied with the reference methodology MR-004 (IRB number: 2022-125).

We finally retained patients for whom the MB harbor (i) a clear PV (class 4 and 5) in *ELP1*,^16^ or (ii) a variant of unknown significance with loss of ELP1 expression in the tumor detected by immunohistochemistry. From the MB samples published in Waszak et al.,^12^ 1 sample (MB07_09) was discarded because the variant was of unknown significance and immunostaining showed a normally retained protein expression.

### MB Molecular Grouping

MB subgrouping between WNT, SHH, group 3, and group 4 was performed by analyzing the expression of 22 selected genes by Nanostring Technology as described previously.^17^ For the 5 patients (MB07_04, MB08_02, MB08_15, MB15_04, and MB15_12), the MB subgroup (between WNT, SHH, G3, and G4) was determined by using Illumina Infinium MethylationEPIC BeadChip arrays as previously reported by Waszak et al.^12^ MB subgroup predictions were obtained from a DNA methylation-based classification web-platform for central nervous system tumors (https://www.molecularneuropathology.org/mnp/, version v12.5).

### Analyses of Other Genes SNV and CNV

Tumor DNA was sequenced with a custom NGS composed of 571 genes of interest in oncology including the following genes: *APC*, *CTNNB1*, *DDX3X*, *ELP1*, *GLI2*, *KDM6A*, *MYC*, *MYCL*, *MYCN*, *PTCH1*, *SMARCA4*, *SMO*, *SUFU*, *TERT*, and *TP53*. The nucleotide sequence (variant calling is performed using Varscan2) as well as the number of copies (deletion and focal amplification) were explored. Briefly, 50 ng of DNA input extracted from frozen or FFPE MB samples, depending on the tumor material available, were used to prepare the library with the Agilent SureSelect XT-HS preparation kit according to the manufacturer protocol, using the design of the 571 genes and an additional backbone of probes across the whole genome with an average resolution of 1 probe every 200 kb. This allows for determining a ploidy and an estimated cellularity, together with a genomic profile spanning every chromosome. The copy number profile for each tumor was estimated using a combination of homemade R scripts and facets package (v0.6.0) with a sex-specific unmatched-germline control previously sequenced using the same panel for normalization. Thirty-two DNA were sequenced per 2 × 100 Sp flowcell of the NovaSeq Sequencer (Illumina) to reach an average depth of 1500× and a minimum depth of 100× on the region of interest.

### Analysis of the Germline DNA

Germline DNAs were obtained from geneticists after informed consent from the parents or legal representatives. Blood samples were extracted on a Qiasymphony with Minikit (Qiagen). The library preparation was on the Agilent SureSelect QXT HS according to the manufacturer protocol on a 100 gene-panel. All 37 exons of the *ELP1* gene (NM_003640) were analyzed to assess genetic variations comprehensively. The coverage of exons and nearby intronic regions within ±50 bp is ensured through Sanger resequencing of exon and nearby intronic regions between –20 and +6 bp, specifically targeting low coverage (<100 X or <30 X for variants exclusively involving single nucleotide substitutions). Sanger resequencing is also conducted for class 4 and 5 variants, with large rearrangements detected using a bioinformatics method (coverage profile). The results are further confirmed through a bioinformatics pipeline, including a web interface for result validation with Grio-Dx v.2.0, and analytical sensitivity calculated during method validation, achieving 100% CI 95% [98.9 – 100] for point variants and CI 95% [97.9 – 100] for large rearrangements. The reference genome used is GRCh37 (hg19), with alignment performed using bwa v.0.7.5a, variant detection using GATK Haplotype v-3.4-46 and home-made programs for automatic reading of BAMs, and variant annotation using snpEff v-5.3.0. Additionally, DBSNP v.b147, Cosmic v69, dbNSFP v2.5, ESP6500SI-V2-SSA137, and ExAC v.r0.3 databases are consulted, followed by in silico verification of variant annotations with Alamut v.2.15, and nomenclature adherence to HGVS ATG 1 (Human Genome Variation Society). Finally, variants involving splicing events were confirmed with RNASeq analysis to validate their deleterious impact. Only class 4 and 5 variants were reported.^16^ A local genetic counseling and germline analysis protocol were used for the Dutch patient.

### Clinical Information

Clinical and molecular data of patients treated in France were collected in the “Observatory of Genetic Cancer Predisposition Syndromes in Children and Adolescents” French database (*Observatoire des syndromes de prédisposition génétique au cancer des enfants et des adolescents*, PREDCAP, IRB00003888). Briefly, age at tumor-onset, metastatic status, local histopathological conclusions, treatments, information on relapse, and last news was recorded by the treating physicians. Patients were treated with a combination of surgery, chemotherapy and radiotherapy according to HIT-SKK,^18^ PNET5 MB,^19^ M-SFOP 1998 or 2007,^20,21^ or PNET HR + 5^22^ protocols for most of them. Survival curves were obtained with the Kaplan–Meier method and using the log-rank test. Information on pedigrees, familial histories of cancer and associated congenital defects in the probands were recorded by local geneticists. In order to assess the penetrance and oncological spectrum of *ELP1* PV, we studied the medical history of the 11 families in which PV was proven to be inherited. We finally added 1 family originating from the Netherlands where 2 cousins were reported with an MB in the context of a confirmed germline *ELP1* PV; the clinical characteristics of MB in this family were not included in the general description of *ELP1* mutated MB treated in France.

## Results

### Molecular Characteristics of ELP1-Mutated MB

**Figure 1.**
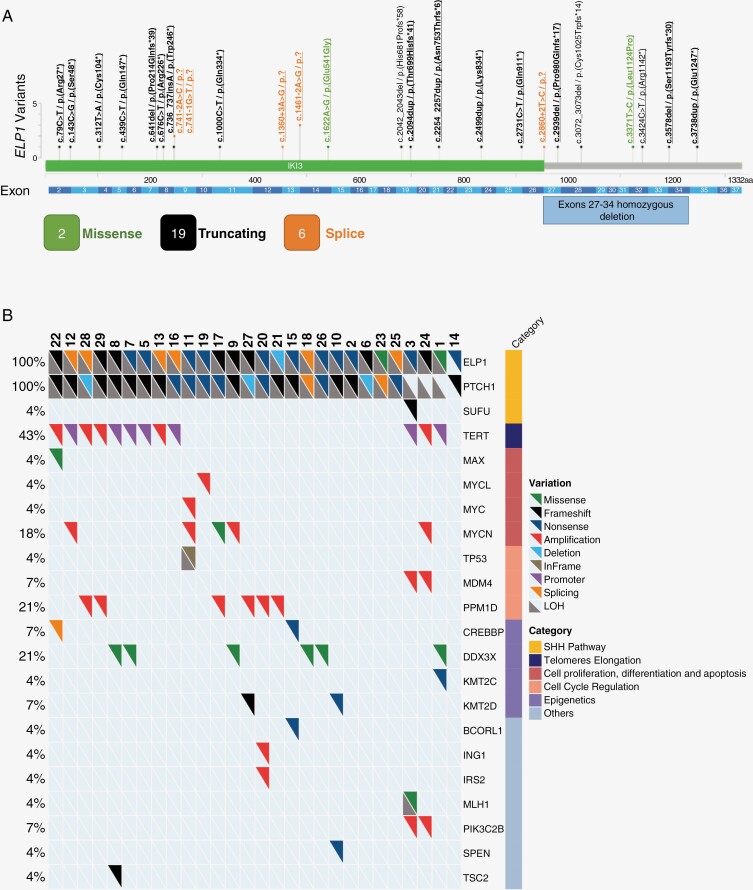
Molecular features of *ELP1*-mutated MB. (A) Summary and gene localization of all pathogenic variants (PV) found in our series of 29 MB. Black color refers to truncating variants, green color to missense variants, and orange color to splice site variants. (B) Oncoprint on the 28 MB samples for which next-generation sequencing was performed: each column refers to a sample, each line to 1 gene; genes are ranked according to the frequency of genetic alterations occurring in the pathway they are involved in. Only ELP1-Fr3 could not be analyzed (sample not available).

We identified 29 patients from 28 families treated in France, who developed an MB harboring an *ELP1* PV (*n* = 27) or a likely pathogenic variant with loss of protein expression (*n* = 2), including 5 patients previously described in the original publication for *ELP1* PV identification (MB07_04, MB08_02, MB08_15, MB15_04, and MB15_12).^12^ A summary of *ELP1* variants is provided in Figure 1A.

As expected, all *ELP1*-mutated MB belonged to the MB-SHH group on immuno-histochemical analyses. The molecular subgroup was confirmed SHH for all 19 samples analyzed (Table 1). In agreement with previous reports, 24/28 informative tumors showed a co-occurring *PTCH1* biallelic inactivation including a large 9q deletion encompassing both *ELP1* and *PTCH1* loci, confirming that the “four hit-three steps” model is the rule for these MB; 1 single *PTCH1* alteration was found for the 4 remaining tumors, one of which also showed a heterozygous *SUFU* inactivating PV; finally, for 1 tumor, *ELP1* sequence and *MYCN* FISH results were the only available biological characteristics. *TERT* over-activation through hotspot promoter mutations (c.-124C > T/p.?) (*n* = 7) or amplification (*n* = 5) was the second most frequent genetic event (43% of cases). The *TP53* pathway was also frequently altered, through *PPM1D* amplification in 6 samples, *MDM4* amplification in 2 samples and *TP53* PV in 1 sample (altogether, 32% of cases). Interestingly also, 4 tumors showed an *MYCN* amplification and 1 an oncogenic *MYCN* missense variant, 1 sample showed an *MYCL* amplification, 1 showed an *MYC* amplification, and 1 a *MAX* hotspot activating mutation; these findings suggest that the *MYC/MYCN/MYCL* signaling is recurrently active in those MB-SHH (28% of cases altogether) (Figure 1B).

**Table 1.** Summary of Clinical Features of the 29 Patients from the French Series Metastatic Status, According to Chang Classification (M0–4)^29^; M + Refers to Metastases, Not Specified. HITSKK,^18^ MSFOP With Hyperfractionated Irradiation,^20,21^ And PNET HR + 5^22^ Protocol Have Been Published.

Patient	*ELP1* Germline Status	Inheritance	Age at Diagnosis of MB (years)	Metastatic Status	MB Histology/Molecular Subgroup	*MYC, MYCN*, or *MYCL* Alteration	*TP53* Pathway Alteration	*TERT* Alteration	Therapeutic Strategy	Relapse (Delay After MB dg)	Statut At last-FU (Delay After MB dg)	Other Malignancy (Delay)
MB07_04	Mut	NA	6 y	M3	DNMB/SHH				PNET HR + 5	No	NED (11.8 y)	
MB08_02	Mut	NA	10 y	M0	DNMB/SHH		*PPM1D* amplification	*TERT* amplification	MSFOP 1998	No	NED (10.8 y)	
MB08_15	Mut	NA	7 y	M0	Classical/SHH		*PPM1D* amplification		MSFOP 2007	No	DOC (5.9 y)	Cerebellar HGG (5.2 y after MB)
MB15_04	Mut	Yes, from mother	8 y	M0	DNMB/ SHH		*PPM1D* amplification	*TERT* amplification	MSFOP 2007	No	NED (7.9 y)	
MB15_12	Mut	NA	5 y	M0	DNMB/SHH				MSFOP 2007	No	NED (4.1 y)	
ELP1-Fr1	Mut	Yes, from mother	7 y	M0	Classical/ NA			*TERT* promoter mutation	PNET 5 MB	No	NED (2.7 y)	
ELP1-Fr2	Mut	Yes, from father	8 y	M2	DNMB/NI		*MDM4* amplification	*TERT* promoter mutation	PNET HR + 5	No	NED (5.5 y)	
ELP1-Fr3	Mut	NA	4 y	M0	DNMB/NA	Not done	Not done	Not done	HITSKK	Yes (1.8 y)	AWD (3.2 y)	
ELP1-Fr4	Mut	Yes, from father	4 y	M0	DNMB/SHH			*TERT* promoter mutation	VPC + HDC	Yes (2.1 y)	DOD (6.5 y)	
ELP1-Fr5^a^	Mut	Yes, from father	7 y	M0	LCA/SHH				PNET HR + 5	No	NED (2.7 y)	
ELP1-Fr6^a^	Mut	Yes, from mother	15 y	M0	DNMB/NA	*MYCN* variant (c.131C > T)	*PPM1D* amplification		MSFOP 1998	No	NED (1.9 y)	
ELP1-Fr7	Mut	NA	14 y	M0	DNMB/NA			*TERT* promoter mutation	VPC + CSI	Yes (1.4 y)	DOD (1.7 y)	préB-ALL (before MB)
ELP1-Fr8	Mut	Yes, from mother	14 y	M0	DNMB/SHH			*TERT* promoter mutation	PNET 5 MB	No	NED (5.5 y)	
ELP1-Fr9	NA	NA	3 y	M0	DNMB/ SHH	*MYCN* amplification			VPC + HDC + focal RT	Yes (0.8 y)	DOD (1.5 y)	
ELP1-Fr10	Mut	Yes, from mother	4 y	M0	DNMB/ SHH				HIT SKK	No	NED (2.8 y)	
ELP1-Fr11	Mut	NA	9 y	M0	LCA/NA	*MYC* and *MYCN* amplification	*TP53* PV (c.754_762del)		PNET HR + 5	Yes (2.1 y)	DOD (4.2 y)	
ELP1-Fr12	Mut	NA	11 y	M0	LCA/SHH	*MYCN* amplification		*TERT* promoter mutation	PNET HR + 5	Yes (3.7 y)	AWD (6.4 y)	
ELP1-Fr13	Mut	NA	14 y	M0	DNMB/SHH			*TERT* amplification	PNET 5 MB	No	NED (1.4 y)	
ELP1-Fr14	Mut	NA	3 y	M0	DNMB/NA				HIT SKK	No	NED (8.1 y)	
ELP1-Fr15	Mut	NA	14 y	M0	DNMB/SHH				HIT SKK	No	NED (1.2 y)	
ELP1-Fr16	Mut	Yes, from father	7 y	M0	DNMB/SHH			*TERT* promoter mutation	MSFOP 2007	Yes (2.4 y)	NED (2.8 y)	
ELP1-Fr17	Mut	NA	9 y	M0	DNMB/SHH				VPC + ICS + CT	No	NED (9.2 y)	
ELP1-Fr18	NA	NA	8 y	M+	NA/SHH	*MYCL* amplification			PNET HR + 5	No	NED (10.7 y)	
ELP1-Fr19	Mut	Yes, from father	5 y	M0	DNMB/SHH		*PPM1D* amplification	*TERT* amplification	VPC + CSI	No	NED (4.0 y)	Thyroid carcinoma (3.1 y after MB)
ELP1-Fr20	Mut	NA	6 y	M0	Classical/ SHH		*PPM1D* amplification		PNET 5MB	No	NED (3.9 y)	
ELP1-Fr21	Mut	NA	13 y	M+	DNMB/SHH	*MAX* mutation		*TERT* amplification	PNET HR + 5	No	NED (2.2 y)	
ELP1-Fr22	Mut	NA	3 y	M0	DNMB/NA				HIT SKK	Yes (1.3 y)	NED (8.0)	
ELP1-Fr23	Mut	Yes, from mother	11 y	M0	LCA/NA	*MYCN* amplification	*MDM4* amplification		PNET HR + 5	No	NED (2.1 y)	
ELP1-Fr24	NA	NA	6 y	M3	DNMB/NA				VPC + ICS + CT	No	NED (16.1 y)	

^a^ELP1-Fr5 and 6 are cousins from the same family.

Abbreviations: ALL: acute lymphoblastic leukemia; AWD: alive with disease; CC: conventional chemotherapy; CSI: cranio-spinal irradiation; DNMB: desmoplastic/nodular medulloblastoma; DOD: dead of disease; FU: follow-up; HDC: high-dose chemotherapy; HGG: high-grade glioma; LCA: anaplastic/large cell; MB: medulloblastoma; Mut: mutated; NA: not available; NED: no evidence of disease; NI: non interpretable; SHH: Sonic Hedgehog; VP–CBP: Vepeside and carboplatine course.

### Clinical Characteristics of ELP1-Mutated MB and Patients’ Outcome

**Figure 2.**
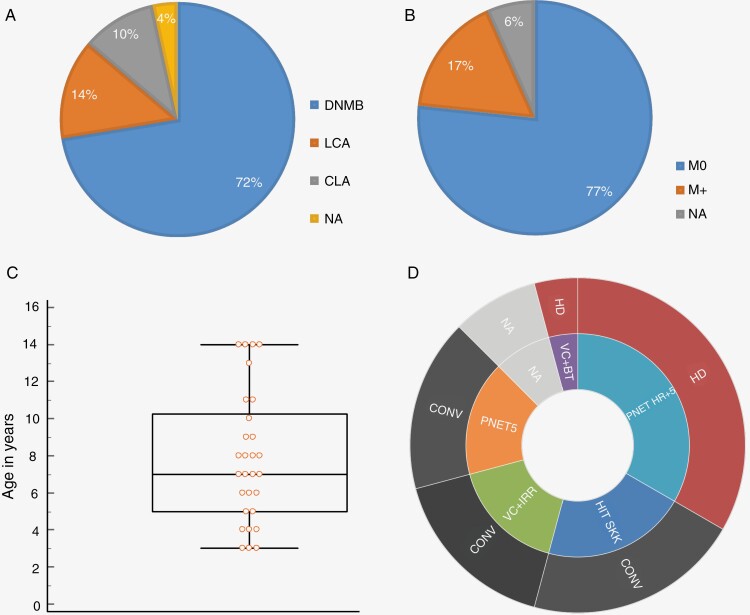
Clinical features of *ELP1*-mutated MB. (A) Repartition of the various histological types (local diagnosis, no central review): large cell/anaplasia containing MB (LCA), classic MB (CLA), and nodular desmoplastic/extensive nodularity MB (DNMB); MB with no available histological type (NA). (B) Repartition of metastatic status within the cohort; M0 to localized disease, and M+ to metastatic cases. NA refers to cas with unknown status. (C) Box-plot showing the distribution of ages of patients, in years, at the time of diagnosis; each dot corresponds to 1 patient’s age. (D) Various treatments administered to patients; in the external circle, conventional chemotherapy (CONV) and high-dose chemotherapy containing regimen (HD); in the internal circle, PNET5 refers to treatment based on the PNET5 SIOPE protocol; PNET HR + 5 refers to treatments based on the French SFCE PNET HR + 5 protocol, HIT-SKK refers to treatments based on the HIT SKK treatment; VC+IRR refers to Vincristine and Irradiation only; VC+BT  refers to VP16-Carboplatin followed by Busulfan-Thiotepa strategy; NA refer to unspecified treatment.

*ELP1*-mutated MB showed various histopathological features according to local pathologist records: nodular desmoplastic ± extensive nodularity (*n* = 21/28, 75%), classic (*n* = 3/28, 11%) or large/cell anaplastic (*n* = 4/28, 14%) (Figure 2A). The tumor was localized for 24/29 patients (83%), and metastatic in 5/29 (Figure 2B). Median age at diagnosis was 7.3 years (range [3–14], Figure 2C).

Patients were treated in 14 different sites from the Société Française de Lutte contre les Cancers et leucémies de l’Enfant et de l’adolescent (SFCE). Given the large period covered by the study and the heterogeneity in ages and risk stratifications, treatments were highly heterogeneous (Table 1, Figure 2D). Briefly, the therapeutic strategies were as follows: according to HIT-SKK protocol^18^(5 patients), PNET5 MB protocol^19^ (4 patients), MSFOP 1998 or 2007 protocol^20,21^ (6 patients), PNET HR + 5 protocol^22^ (8 patients), regimen comprising conventional chemotherapy, and craniospinal radiotherapy (4 patients) and regimen comprising conventional and high-dose chemotherapy ± followed by focal radiotherapy (2 patients). Regarding the treatment-associated adverse effects, no short-term unexpected toxicity was recorded for these patients.

**Figure 3.**
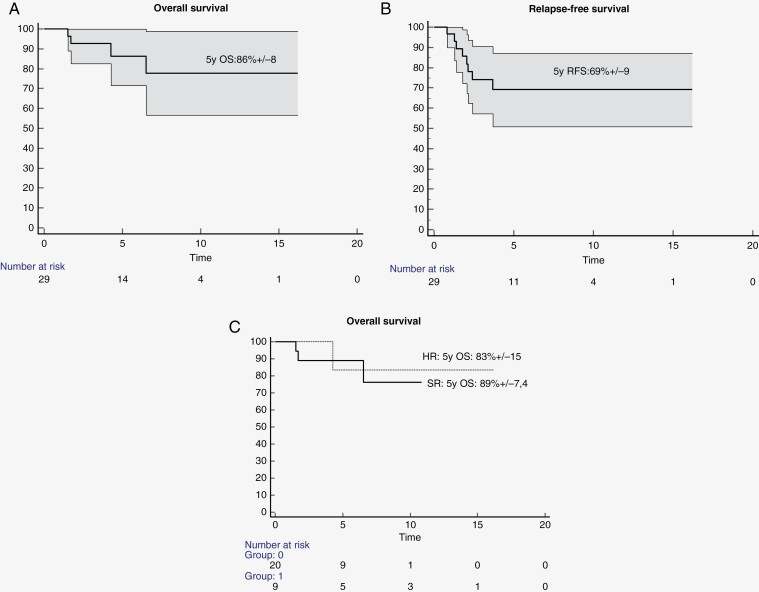
Patient outcomes assessed by the Kaplan–Meier method. (A) Overall survival of the entire cohort of patients affected by MB: *x* axis refers to the time from diagnosis in years, and *y* axis refers to the actualized percentage of alive patients. (B) Relapse-free survival in the entire cohort. (C) Overall surviving according to risk factors, HR refers to the high-risk in thin line (group 1, including LCA, M+, TP53 mutated, or MYCN amplified MB), and SR refers to low to intermediate risk in thick line (group 0, all others). Numbers at risk are specified below the *x* axis.

Median age at last follow-up was 13 years [range 5–22]. The 5-year overall and relapse-free survival were 86 ± 7.9% and 69 ± 9.2%, respectively (Figure 3A and B). Overall, 24 (83%) children were alive with a median follow-up from diagnosis of 4.0 years (range [1.2–16.1]). Among the 5 deceased patients, 4 patients died due to MB progression (including 1 with the somatic *TP53* PV) (median time since diagnosis: 1.7 years, range [0.8–2.1]) and 1 because of secondary cancer arising in the irradiation field (malignant high-grade glioma). There was no statistical difference between high-risk and standard-risk MBs in this series (Figure 3C), in line with risk-adapted treatments reported in this cohort.

### Germline DNA Analysis and Genetic Inheritance

We could sequence the germline DNA in 26/29 (90%) patients with *ELP*1-mutated MB. Strikingly, the homozygous PV found in the tumor was retrieved heterozygous at the germline level in 26/26 cases (100%). The heterozygous *ELP1* PV coexisted with a heterozygous MLH1 PV in the germline in 1 patient (ELP1-Fr2). No remarkable congenital malformation nor intellectual disability was reported to be associated with MB in our series.

**Figure 4.**
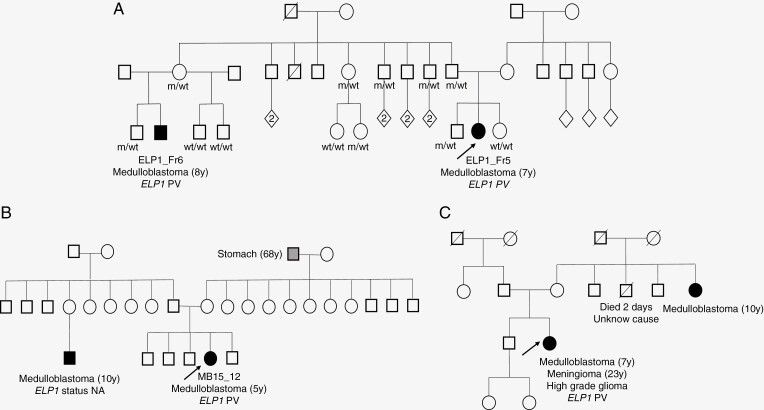
Pedigrees of familial cases. (A) Pedigree of the family of patients ELP1-Fr5 and ELP1-Fr6. (B) Pedigree of the family of patient MB15_12. (C) Pedigree of the Dutch family. The index case is pointed to by an arrow; black box refers to MB; wt: wild-type; PV: pathogenic variant; NA: not assessed.

We then sequenced germline DNA from 11 trios (cas index and his parents) in order to estimate the rate of de novo versus inherited PV. Strikingly again, 11/11 trio analyses revealed that the PV was inherited from an asymptomatic parent (6 mothers and 5 fathers). A familial history of MB was found in only 1 of these 11 families: as depicted in the pedigree in Figure 4A, 1 second-cousin (ELP1-Fr6) of the proband (ELP1-Fr5) was a carrier of the familial PV and also affected by an MB, but many asymptomatic carriers were identified in the family, including a majority of adults beyond the upper age of tumor-onset observed in our series. In addition, 1 patient with MB and *ELP1* PV (patient MB15_12) but with no DNA available from any relatives, also had a familial history of MB in a first-cousin affected at the age of 10 years (Figure 4B). Finally, we added data from 1 Dutch family; as shown in the pedigree (Figure 4C), her maternal aunt was diagnosed with an MB at the age of 10 years; other familial data were not precise enough to be reported here.

### Secondary Malignancies in Patients With Germline PV in ELP1

We then wondered whether patients with *ELP1* variants were exposed to other malignancies. Among children presenting an MB, we found 5 patients of our French series affected by multiple neoplasms: (i) 1 patient (*ELP1*-Fr7) was first diagnosed with a preB-ALL 18 months before the occurrence of the MB; no 9q loss was found in the blasts, rather suggesting an *ELP1*-independent neoplasm, (ii) 1 patient (ELP1-Fr19) developed an invasive papillary thyroid carcinoma (without loss of ELP1 expression in the tumor sample) at the age 9 years (2.6 years after the end of the craniospinal irradiation, median dose to thyroid = 25.4 Gy), (iii) 1 patient (ELP1-Du1) developed a meningioma at the age of 23, 16 years after the end of irradiation, within the irradiated field, a unilateral ovarian borderline serous tumor at 30 years, and a benign thyroid nodule at 32 years, and (iv) 3 patients (MB08_15, ELP1-Fr18 and ELP1-Du1) were diagnosed with a malignant high-grade glioma, 5.2 years, 10.7 years, and 25 years after MB diagnosis, within the irradiated field. The secondary HGG was analyzed for patient ELP1-Fr18 and ELP1 protein expression was normally retained. The delays between the second malignancy and irradiation, and the expected histology for a radiation-induced tumor with normal expression of ELP1 protein were rather compatible with a radiation-induced tumor. Furthermore, none of the index cases’ parents carrying the variant (aged between 30 and 60 years) were declared to be affected by any neoplasm at the time of the genetic consultation. Altogether, our observations rather suggest a tumor spectrum restricted to childhood MB, and the tumor-free survival in *ELP1-*variant carriers (Figure 3C) indicates that the risk may be limited to the first 15 years of life.

## Discussion

We previously reported that ELP1 immunostaining in MB is a very efficient tool for predicting the presence of a PV in the tumor.^15^ We now emphasize that the presence of an *ELP1* PV in tumor DNA is always correlated with its presence also at the germline level, thus systematically leading to the diagnosis of CPS.

Once a CPS has been diagnosed, one of the first issues to be addressed is whether therapeutic strategies should be adapted to the genetic background, depending on the prognosis of MB and the risk of a second malignancy. This has been largely discussed for patients baring *TP53, PTCH1 or SUFU* constitutional PV.^9,11^ Here we show that: (i) *ELP1* PV predispose to SHH-MB of any risk group, (ii) the prognosis of *ELP1*-associated MBs is similar to those of sporadic cases with the same risk factors, and (iii) finally, no short-term unexpected or remarkable toxicity was retrospectively recorded using classical treatments. Among the 5/30 patients (16.7%) presenting more than 1 malignancy, 4 developed secondary cancer in the radiation fields after craniospinal irradiation, compatible with histologies and delays observed in patients with sporadic MB after similar treatments, as reported elsewhere.^23,24^ The last extra-MB malignancy on our series (preB-ALL) preceded the treatment; its relation to *ELP1* PV is not obvious given the lack of *ELP1* PV reported in pediatric hemopathies so far, and the absence of 9q loss. Altogether, our data don’t support any particular adaptation of the treatments for *ELP1*-mutated MB, and we believe that these patients should thus be treated as sporadic cases with similar risk factors. Nevertheless, our cohort is small and a careful follow-up of second malignancies in *ELP1* PV carriers is still needed.

Remarkably, the family studies showed that *ELP1* VPs were inherited from an asymptomatic parent in all analyzed cases, equally from mothers or fathers. A second history of MB was found only in 3 large families, and then only in aunts or cousins, none in siblings. These 3 pedigrees and those of the families analyzed in trio suggest that many carriers remain unaffected beyond the median age of MB onset. The lack of comprehensive analyses of all relatives in those families precludes a definitely reliable estimation of the penetrance. However, the pedigrees show that relatives of patients with *ELP1*-related MB have a low risk of developing cancer, which highlights that the penetrance is highly incomplete. Likewise, in a recent article, Smith et al. reported that *ELP1* loss-of-function (LoF) variants are frequent in the gnomAD population data (close to 1 in 1000), leading to a risk of developing an MB less than 1% in carriers of *ELP1* PV out of any familial context.^25^ Of note, this low risk of MB is broadly similar to that related to *PTCH1* PV, for which no screening for MB in childhood is recommended.^6,26^ Whether the occurrence of an MB in families harboring an *ELP1* PV results from additional germline genetic modifications remains speculative, and obviously not accessible to any investigation of clinical use so far. In that context, Smith et al. proposed no radiological screening for MB in individuals with incidental findings of an *ELP1* PV; but these authors still questioned the relevance of closer surveillance in relatives of patients with MB.^25^ Given the low penetrance, the restricted spectrum and the harmful stress resulting from excessive surveillance, the benefit of testing the *ELP1* gene for these index patients with an MB is disputable. In 1 part, we would still consider it relevant to propose genetic counseling for any patient with MB in order: (i) to propose to shed some light on a genetic cause for the MB occurrence, which might help some parents in their understanding of the disease^27^ and (ii) to raise awareness about symptoms that may lead to earlier clinical investigations in relatives in the “at risk” age range. However, this must be balanced with the psychological burden of genetic analysis and the lack of clear evidence for proposing surveillance of asymptomatic *ELP1*-PV carriers. Of note, the surveillance period would need to cover about 10 years (5–15 years of age), a much longer period than what is currently recommended for individuals with a *SUFU* PV (first 5 years of life only), while the penetrance seems to be far less. Therefore, the psychological impact really needs to be taken into account to avoid excessive distress among parents when revealing an underlying cancer predisposition among their affected children.^28^ Our results thus suggest that genetic testing should not be routinely nor systematically offered to relatives, and should probably be preferentially performed as part of dedicated research programs. Anyway, the level of uncertainty raised by our study stresses the need for psychological support if a genetic survey is decided.

Given their low penetrance, *ELP1* PVs could be considered at the edge between susceptibility and true predisposition; however, the overall risk of developing an MB is still much higher in *ELP1* PV carriers than in the general population (RR: 33),^25^ which clearly suggests an actual oncogenic role for *ELP1* LoF in MB oncogenesis. Our series confirms that *ELP1* LoF acts in synergy with *PTCH1* loss of function, as a key and constant step in those malignancies; the exact interplay between *ELP1* and SHH-pathway deregulation remains to be elucidated. One could speculate that the *ELP1* inactivation increases the risk of *PTCH1* inactivation, or increases the number of cells likely to be transformed upon SHH over-activation. Furthermore, the genetic analyses we carried out on our series of MB showed that additional events such as *MYC/MYCN/MYCL* signaling activation, *TP53* function deregulation (through *TP53* PV, *MDM4* amplification or *PPMI1D* amplification) and *TERT* overactivity may also bring some oncogenic advantage to *ELP1*-mutated cells, in addition to *PTCH1* LoF. Of note, the mutual exclusivity of *TP53* deregulation and *ELP1* LoF is less obvious in our series than previously reported. Finally, mouse models taking into account these various genetic alterations in an *ELP1*-mutated context may help better understand the actual role of *ELP1* in MB oncogenesis and guide practitioners towards potential targeted therapies.

*ELP1*-mutated MB do not appear to differ from sporadic MB-SHH in terms of clinical features, outcome and subsequent oncological risk. Our study shows that an *ELP1* germline PV is found in 100% of cases when ELP1 expression is lost on immunostaining and/or *ELP1* somatic mutation is reported, and that the PV is always inherited when a family genetic analysis is available. Our study reports no other cancer risk than MB risk associated with *ELP1*. Moreover, in this entity, *ELP1* appears to be an oncogenic driver, but not the only 1. Given the low risk of MB without additional malignancies in *ELP1* PV carriers, our study questions the actual benefit of genetic screening in these patients and their family and suggests restricting *ELP1* germline sequencing to patients with MB, depending on the parents’ demand.

## Data Availability

Sequencing data are available on request.
